# Optimization of factors influencing exopolysaccharide production by *Halomonas xianhensis* SUR308 under batch culture

**DOI:** 10.3934/microbiol.2017.3.564

**Published:** 2017-07-06

**Authors:** Jhuma Biswas, Amal K. Paul

**Affiliations:** Microbiology Laboratory, Department of Botany, University of Calcutta, Kolkata-700019, West Bengal, India

**Keywords:** *Halomonas xianhensis*, solar salterns, moderately halophilic bacterium, exopolysaccharides, hydrocarbons

## Abstract

A moderately halophilic bacterium, *Halomonas xianhensis* SUR308 (GenBank Accession No. KJ933394) was isolated from multi-pond solar salterns of Odisha, India. Exopolysaccharide (EPS) production by this strain in malt extract yeast extract (MY) medium has been optimized under batch culture system. Among the different media tested, MY medium showed an EPS production of 2.55 g/L, which increased to 2.85 g/L under optimized aeration. An initial pH of 7.5 and incubation temperature of 32 °C were found to be most suitable for EPS production by the isolate under aerobic condition. An EPS production of 3.85 g/L was achieved when the growth medium was supplemented with 2.5% NaCl. Glucose was the most favourable carbon source for EPS production and maximum production (5.70 g/L) was recorded with 3% glucose. However, growth as well as production of EPS was remarkably affected when the growth medium was supplemented with hydrocarbons as sole source of carbon. Among different nitrogen sources, casein hydrolysate at 0.5% level was proved to be the best for EPS production and an initial inoculum dose of 7% (v/v) enhanced the EPS production to 7.78 g/L, while the divalent metal ions were in general toxic to growth and EPS production, EPS synthesis by SUR308 was enhanced with Cr (VI) supplementation.

## 1. Introduction

In recent years, increasing attention is being paid to microbial exopolysaccharides (EPS) mainly because of their bioactive role and extensive range of potential applications in modern biotechnology especially in medicine and pharmaceuticals as antiangiogenic or antiviral agents or even in case of targeted drug delivery [1]. The advantages of microbial exopolysaccharide over plant and marine micro-algal biopolymers are due to their novel functionality, easily reproducible chemical and physical properties, and stable cost and supply. Moreover, EPS from halophiles have stability towards higher temperature, salinity and even pH [2]. These unique properties of EPS produced by halophiles seem to offer diverse applications in various fields of industry [3].

Halophilic bacterial strains have been reported to produce exopolysaccharide (EPS) in their natural hypersaline environments as well as under laboratory cultural conditions. Among the wide diversity of halophilic species, members belonging to the moderately halophilic genus *Halomonas* have been found to produce EPS in a technologically viable way. Polysaccharides produced by *Halomonas eurihalina* [4], *H. maura* [5] have been described and well characterized. Aqueous solution of EPS synthesized by *H. eurihalina* has been reported to jellify at acidic pH, while mauran, an anionic, sulfated EPS with high uronic acid composition produced by *H. maura* are being used for different biotechnological purposes [6],[7]. The extracellular polysaccharide production by *H. ventosae, H. anticariensis* and *H. almeriensis* [8] has been optimized with a view to identify their components and physico-chemical properties [9]. More recently, several others [10],[11] have also documented EPS production from a number of novel *Halomonas* spp. isolated from diverse ecological conditions.

Production and characterization of exopolysaccharide of *H. xianhensis* SUR308 (GenBank Accession No. KJ933394) has been reported recently [12],[13]. The present study was conducted to optimize the medium components and other environmental parameters for EPS production by *H. xianhensis* SUR308 under batch cultivation. For better understanding of the process, the influence of aeration, pH, temperature, NaCl, carbon and nitrogen sources, inoculum dose along with metal ions on the EPS production by the isolate SUR308 have been evaluated.

## 2. Materials and Method

### 2.1. Bacterial strain and growth condition

*Halomonas xianhensis* SUR308 (GenBank Accession No. KJ933394), a potent EPS producing moderately halophilic bacterium isolated from solar salterns of Surala, Odisha, India was used throughout this study. The isolate was maintained on slopes of MH agar medium [14] by subculturing at a regular interval of one month and stored at 4 °C as and when required.

### 2.2. Production of EPS by *H. xianhensis* SUR308

Microbial growth and EPS production by the isolate were monitored in modified MY medium [4] under batch culture. The MY medium contained (g/L) NaCl, 50; MgCl_2_·6H_2_O, 9; MgSO_4_·7H_2_O_,_ 13; CaCl_2_·2H_2_O, 0.2; KCl, 1.3; NaHCO_3_, 0.05; NaBr, 0.15; FeCl_3_·6H_2_O, 0.005; glucose, 10; yeast extract, 3; malt extract, 3; protease peptone, 5 (pH 7.2). *H. xianhensis* SUR308 was grown in MY medium for 24 h at 32 °C under continuous shaking and used as the inoculum. The medium (20 mL/100 mL flask) was inoculated at 2% level with the fresly prepared inoculum and incubated at 32 °C under continuous shaking for 11 days. To establish conditions leading to the maximum EPS production, the isolate was grown under different cultural variables such as aeration, pH, temperature, carbon source, nitrogen source, NaCl concentration, glucose concentration, hydrocarbons and heavy metals. Each experiment was conducted in triplicates and the average ± SE was recorded.

### 2.3. Measurement of growth

Growth of the isolate SUR308 was determined by measuring optical density (OD) at 540 nm using an ELICO (CL 157) colorimeter. To determine the dry cell weight (DCW), known volume of culture was centrifuged at 5000 × g for 10 min and the cell mass after thorough washing was transferred into a pre-weighed aluminum foil cup. The cell mass was then dried to constant weight at 80 °C and the difference in weight was used to represent the actual dry weight.

### 2.4. Isolation and estimation of EPS

The EPS content of the growing culture was isolated and quantified according to the method as described by Quesada et al. [4]. To extract the soluble EPS, the culture after definite period of growth was centrifuged at 12,000 × g for 30 min, three volumes of chilled ethanol was added to the supernatant and kept overnight at 4 °C for precipitation of the EPS. The precipitate was then collected by centrifugation at 12000 × g for 30 min. The cell bound EPS was extracted from the cell mass by treating with hot normal saline for 10 min under vigorous shaking. It was re-centrifuged at 10,000 × g for 15 min to separate the cell mass. The EPS in the supernatant was recovered as per the method of soluble EPS extraction described above. The soluble and cell bound EPS were pooled, dissolved in known volume of water and quantified for total carbohydrate.

The carbohydrate content was quantified by Dubois method [15]. To 1 mL of EPS solution, 0.5 mL of 5% phenol and 3.5 mL of concentrated sulfuric acid was added and incubated at 50 °C for 20 min. Absorbance was recorded at 490 nm and the concentration was determined from the calibration curve prepared in the same method using glucose as standard. As and when required the glucose content in the culture was estimated by dinitro salicylic acid (DNS) method as described by Miller [16].

## 3. Results and Discussion

### 3.1. Selection of culture medium

For screening of culture media suitable for EPS production, the isolate was grown in media of 14 different types supplemented with 5% NaCl. Growth and EPS production were estimated after 11 days of growth under continuous shaking at 32 °C. Results represented in Table 1, indicated that complex media such as malt extract-yeast extract medium (MY), medium for halophiles (MH), medium A, medium C, medium 1 and tryptone yeast extract medium favored the production of EPS by the isolate compared to the chemically defined media used. Among the complex media, MH medium showed maximum growth, while MY medium supported the growth associated EPS production (2.58 g/L of EPS).

**Table 1.** Screening of culture media for growth and EPS production by *H. xianhensis* SUR308.

Medium	Growth		Production of EPS, g/L	
	OD at 540 nm	DCW, g/L		
Malt extract-Yeast extract (MY)	4.70 ± 0.20	2.36 ± 0.01	2.58 ± 0.11	
Medium for Halophiles (MH)	5.58 ± 0.24	3.06 ± 0.06	1.44 ± 0.09	
Medium A (Poli et al. 2007)	3.01 ± 0.01	1.18 ± 0.01	1.55 ± 0.04	
Medium B (Poli et al. 2007)	2.01 ± 0.02	0.94 ± 0.11	0.36 ± 0.06	
Medium C (Poli et al. 2007)	4.00 ± 0.04	1.86 ± 0.01	1.41 ± 0.09	
Medium D (Poli et al. 2007)	1.09 ± 0.00	0.41 ± 0.03	0.30 ± 0.04	
Medium 1 (Romano et al. 2007)	4.92 ± 0.07	2.58 ± 0.02	1.55 ± 0.01	
Tryptone-Yeast extract medium (TY)	5.85 ± 0.04	3.25 ± 0.03	1.22 ± 0.07	
Modified Nutrient Broth (NB)	1.32 ± 0.00	0.78 ± 0.08	0.86 ± 0.07	
Zobell's Marine broth (MB)	1.32 ± 0.12	0.62 ± 0.02	0.81 ± 0.04	
Davis Mingioli's (DM)	4.98 ± 0.24	2.05 ± 0.07	0.95 ± 0.06	
Basal medium for halophiles (BH)	0.80 ± 0.01	0.24 ± 0.01	0.20 ± 0.01	
Basal MH medium (BMH)	0.90 ± 0.01	0.70 ± 0.01	0.40 ± 0.02	
Valera synthetic medium	0.47 ± 0.05	0.18 ± 0.01	0.12 ± 0.05	

Fermentations were carried out in MY medium [(g/L): NaCl, 50; MgCl_2_·6H_2_O, 9; MgSO_4_·7H_2_O_,_ 13; CaCl_2_·2H_2_O^·^, 0.2; KCl, 1.3; NaHCO_3_, 0.05; NaBr, 0.15; FeCl_3_·6H_2_O, 0.005; glucose, 10; yeast extract, 3; malt extract, 3; protease peptone, 5; pH 7.2] inoculated with 2% (v/v) inoculum and incubated under continuous shaking (120 rpm) at 32 °C. All values were taken in triplicates and the average ± SE was recorded after 11 days of incubation. One way ANOVA has been carried out for each row with the P value of 0.05. Bonferroni's post-test shows the production of EPS and DCW were significantly different (*P* < 0.05) against growth (OD at 540 nm) while EPS production against DCW was not significant (*P* > 0.05).

Amjres et al. [10] revealed similar influence of cultural and environmental factors on haloglycan production by *H. stenophila* HK30. Similar observation was reported with *H. eurihalina* [4], which produced 2.80 g/L of EPS in MY medium at the stationary phase. Chemical composition of the EPS produced by *H. eurihalina* was depended upon the culture medium used [17]. However, *H maura, H. anticariensis and H. ventosae* produced 4.28, 0.28 and 0.28 g/L of EPS respectively after 5 days of incubation [5],[8].

### 3.2. Effect of aeration

Aeration is another important environmental parameter for production of both intra- and extracellular metabolites of aerobic heterotrophic organisms. The effect of culture volume on metabolite biosynthesis under shake flask fermentation was reflected in the O_2_ concentration of conical flask [18]. As an aerobic heterotrophic bacterium, the strain *H. xianhensis* SUR308 required adequate O_2_ supply for the maximum production of EPS as pointed out by Rehm [19]. To achieve different levels of aeration, the culture volume: flask volume (CVF) ratios were maintained in the range of 1:10–5:10. The production of EPS by the isolate showed an increase when the CVF ratio was changed to 1.5:10 (15 mL/100 mL flask) from 1:10 and attained a maximum EPS production of 2.85 g/L. Following this, when the level of aeration was decreased by increasing the culture volume/flask volume, the EPS production decreased significantly (Table 2). The growth pattern of the isolate SUR308 also suggests its aerotolerant nature.

**Table 2.** Effect of aeration on growth and EPS production by *H. xianhensis* SUR308.

Culture volume: flask volume ratio (CVF)	Growth		Production of EPS, g/L
	OD at 540 nm	DCW, g/L	
1.0:10	3.82 ± 0.11	1.52 ± 0.02	2.48 ± 0.03
1.5:10	4.28 ± 0.02	1.36 ± 0.03	2.85 ± 0.04
2.0:10	4.10 ± 0.08	1.44 ± 0.07	2.66 ± 0.04
2.5:10	4.12 ± 0.04	1.52 ± 0.03	2.62 ± 0.02
3.0:10	3.04 ± 0.08	1.81 ± 0.01	1.89 ± 0.02
3.5:10	3.02 ± 0.06	1.89 ± 0.02	1.76 ± 0.02
4.0:10	2.96 ± 0.06	1.76 ± 0.03	1.67 ± 0.02
5.0:10	2.88 ± 0.04	1.69 ± 0.02	1.56 ± 0.02

Fermentations were carried out in MY medium [(g/L): NaCl, 50; MgCl_2_·6H_2_O, 9; MgSO_4_·7H_2_O_,_ 13; CaCl_2_·2H_2_O, 0.2; KCl, 1.3; NaHCO_3_, 0.05; NaBr, 0.15; FeCl_3_·6H_2_O, 0.005; glucose, 10; yeast extract, 3; malt extract, 3; protease peptone, 5; pH 7.2] inoculated with 2% (v/v) inoculum and incubated at different aeration rates (culture volume:flak volume 1:10–5:10) under continuous shaking (120 rpm) at 32 °C. All values were taken in triplicates and the average ± SE was recorded after 11 days of incubation. One way ANOVA has been carried out for each row with the P value of 0.05. Bonferroni's post-test shows the production of EPS and DCW were significantly different (*P* < 0.05) against growth (OD at 540 nm) while EPS production against DCW was not significant (*P* > 0.05).

### 3.3. Influence of initial pH and temperature

**Figure 1.**
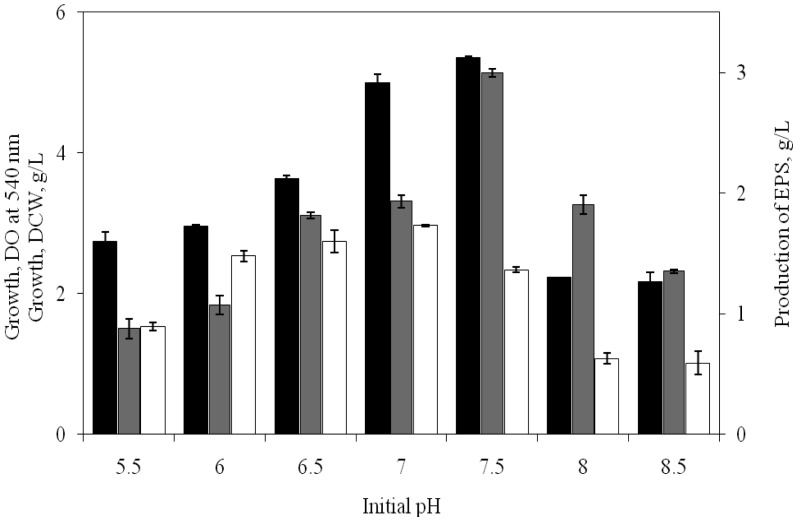
Effect of initial pH on growth [OD at 540 nm (▪), DCW (□)] and EPS production (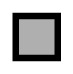) by *H. xianhensis* SUR 308. Fermentations were carried out in MY medium [(g/L): NaCl, 50; MgCl_2_·6H_2_O, 9; MgSO_4_·7H_2_O_,_ 13; CaCl_2_·2H_2_O, 0.2; KCl, 1.3; NaHCO_3_, 0.05; NaBr, 0.15; FeCl_3_·6H_2_O, 0.005; glucose, 10; yeast extract, 3; malt extract, 3; protease peptone, 5] under continuous shaking (120 rpm) at different initial pH ranging from 5.5–8.5 and temperature at 32 °C with 2% (v/v) initial inoculum. All values were taken in triplicates and the average ± SE was recorded after 11 days of incubation.

It was observed from Figure 1 that growth as well as EPS production was increased significantly until the initial pH of the medium reached at 7.5. At this initial pH the amount of EPS production was found to be 2.99 g/L, which remarkably ceased when the medium turned alkaline.

**Figure 2.**
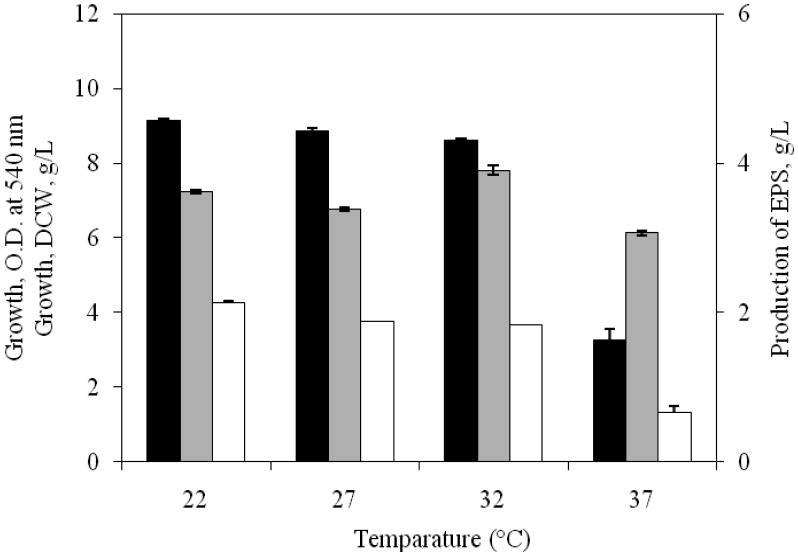
Effect of temperature on growth [OD at 540 nm (▪), DCW (□)] and EPS production (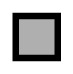) by *H. xianhensis* SUR 308. Fermentations were carried out in MY medium [(g/L): NaCl, 50; MgCl_2_·6H_2_O, 9; MgSO_4_·7H_2_O_,_ 13; CaCl_2_·2H_2_O, 0.2; KCl, 1.3; NaHCO_3_, 0.05; NaBr, 0.15; FeCl_3_·6H_2_O, 0.005; glucose, 10; yeast extract, 3; malt extract, 3; protease peptone, 5] under continuous shaking (120 rpm) at different temperature of 22–37 °C and initial pH 7.2 and with 2% (v/v) initial inoculum. All values were taken in triplicates and the average ± SE was recorded after 11 days of incubation.

At a temperature range of 22–32 °C the isolate was found to produce more EPS along with enhanced biomass production (Figure 2) compared to those at 37 °C. At 32 °C, the isolate showed maximum EPS production (3.50 g/L) and this incubation temperature was retained for subsequent studies.

Thus the influence of pH and temperature on the growth and production of EPS by the isolate SUR308 clearly revealed its neutrophilic and mesophilic nature and supported the earlier works of Quesada et al. [4], Arias et al. [20] and Mata et al. [9] with respect to number of *Halomonas* spp.

### 3.4. Influence of NaCl concentration

**Figure 3.**
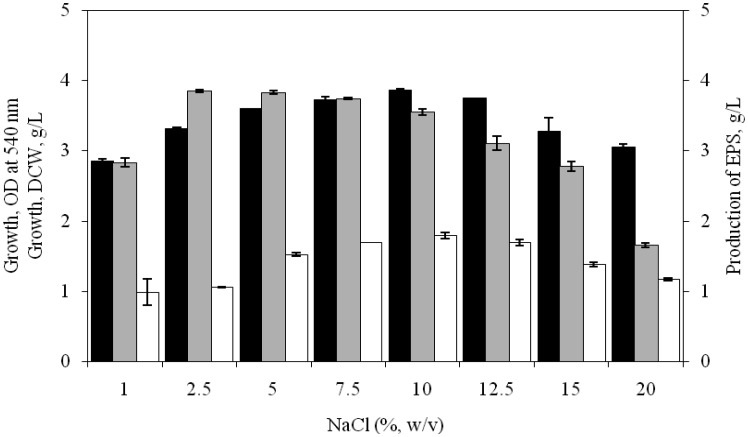
Influence of NaCl concentration on growth [OD at 540 nm (▪), DCW (□)] and EPS production (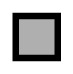) by *H. xianhensis* SUR308. Fermentations were carried out in MY medium [(g/L): MgCl_2_·6H_2_O, 9; MgSO_4_·7H_2_O_,_ 13; CaCl_2_·2H_2_O, 0.2; KCl, 1.3; NaHCO_3_, 0.05; NaBr, 0.15; FeCl_3_·6H_2_O, 0.005; glucose, 10; yeast extract, 3; malt extract, 3; protease peptone, 5] under continuous shaking (120 rpm) at NaCl concentrations ranging from 1–20% (w/v) and initial pH and temperature of 7.2 and 32 °C with 2% (v/v) initial inoculum. All values were taken in triplicates and the average ± SE was recorded after 11 days of incubation.

As a halophilic organism, the isolate SUR308 showed a wide degree of tolerance to NaCl for growth and EPS production was more or less constant in the range of 2.5 to 10.0% NaCl in the medium, where by EPS production varied from 3.85 to 3.54 g/L. It was evident from the results (Figure 3) that the production of EPS was maximum (3.85 g/L) at 2.5% NaCl. Similarly, Arias et al. [20] reported that optimum salt concentrations for EPS production by *H. maura* was 2.5%, while 7.5% salt concentration was proved to be the best for maximum EPS production by *H. eurihalina, H. ventosae* and *H. anticariensis*.

### 3.5. Effect of carbon source

**Figure 4.**
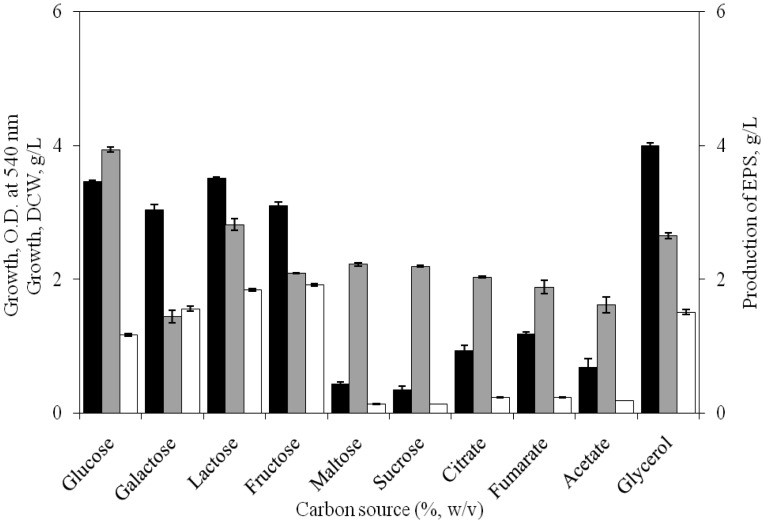
Effect of different carbon sources on growth [OD at 540 nm (▪), DCW (□)] and EPS production (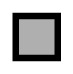) by *H. xianhensis* SUR308. Fermentations were carried out in MY medium [(g/L): NaCl, 25; MgCl_2_·6H_2_O, 9; MgSO_4_·7H_2_O, 13; CaCl_2_·2H_2_O, 0.2; KCl, 1.3; NaHCO_3_, 0.05; NaBr, 0.15; FeCl_3_·6H_2_O, 0.005; yeast extract, 3; malt extract, 3; protease peptone, 5] supplemented with 1% carbon source under continuous shaking (120 rpm) at 32 °C and pH 7.2 with 2% (v/v) initial inoculum. All values were taken in triplicates and the average ± SE was recorded after 11 days of incubation.

The effect of different carbon sources on growth and EPS production by *H. xianhensis* SUR308 revealed that addition of glucose, fructose, lactose or glycerol in the medium at 1% level resulted different levels of growth and EPS production by the bacterium. Glucose was most effective in EPS production (3.95 g/L) followed by lactose (2.82 g/L) and glycerol (2.64 g/L). However, compared to glucose and lactose, glycerol exerted maximum influence on the growth of the isolate. Carbon sources like galactose, acetate, fumarate, citrate along with fructose, maltose and sucrose produced poor to moderate EPS, while fructose and galactose were effectively utilized for growth (Figure 4).

**Figure 5.**
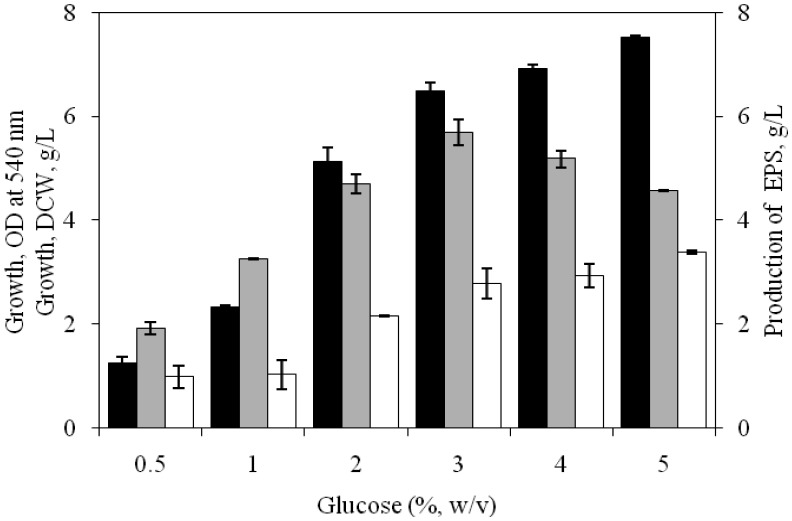
Effect of different concentrations of glucose on growth [OD at 540 nm (▪), DCW (□)] and EPS production (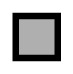) by *H. xianhensis* SUR308. Fermentations were carried out in MY medium [(g/L): NaCl, 25; MgCl_2_·6H_2_O, 9; MgSO_4_·7H_2_O, 13; CaCl_2_·2H_2_O, 0.2; KCl, 1.3; NaHCO_3_, 0.05; NaBr, 0.15; FeCl_3_·6H_2_O, 0.005; yeast extract, 3; malt extract, 3; protease peptone, 5] under continuous shaking (120 rpm) at different concentrations of glucose (5–50 g/L) at 32 °C and pH 7.2 with 2% (v/v) initial inoculum. All values were taken in triplicates and the average ± SE was recorded after 11 days of incubation.

As shown in Figure 5, EPS production was enhanced gradually till the glucose concentration in the medium reached at 3% (w/v), producing 5.7 g/L of EPS. Glucose has been reported to influence EPS production in a number of bacterial species including that of *H. xianhensis* SUR308 but in a number *Halomonas* spp, glucose at >1.0% level retarded the EPS production [4],[8],[9],[20]. Further increase in glucose (4–5%) in the medium retarded the EPS production by the isolate SUR308.

When the influence of different hydrocarbons as sole source of carbon (3%, w/v) was tested on growth and EPS production by the isolate, it was evident that the biomass production as well as the EPS accumulation was remarkably ceased with hydrocarbon supplementation in the medium. When the influence of different hydrocarbons as sole source of carbon (3%, w/v) was tested on growth and EPS production by the isolate, it was evident (Table 3) that the biomass production as well as the EPS accumulation was remarkably ceased with hydrocarbon supplementation in the medium. However, exceptionally hexane and olive oil supported the EPS production (2.97 and 2.64 g/L). This observation was justified by the similar study made on *H. eurihalina* by Martinez-Checa et al. [21].

**Table 3.** Effect of hydrocarbons on growth and EPS production by *H. xianhensis* SUR308.

Hydrocarbons	Growth		Production of EPS (g/L)
	OD at 540 nm	DCW, g/L	
Olive oil	4.84 ± 0.09	1.89 ± 0.01	2.64 ± 0.00
Hexane	5.89 ± 0.09	1.51 ± 0.09	2.97 ± 0.00
Benzene	–	–	–
Tetradecane	2.19 ± 0.09	0.75 ± 0.03	1.06 ± 0.04
Hexadecane	1.71 ± 0.06	0.62 ± 0.09	0.95 ± 0.02
Octane	2.26 ± 0.06	1.09 ± 0.01	0.90 ± 0.11
Diesel	1.93 ± 0.03	0.78 ± 0.05	0.66 ± 0.03
Xylene	–	–	–
Petrol	–	–	–
Kerosene	2.09 ± 0.00	0.68 ± 0.07	0.74 ± 0.02
Castor oil	–	–	–
Control	6.04 ± 0.04	2.88 ± 0.08	5.61 ± 0.01

Fermentations were carried out in MY medium [(g/L): NaCl, 25; MgCl_2_·6H_2_O, 9; MgSO_4_·7H_2_O, 13; CaCl_2_·2H_2_O, 0.2; KCl, 1.3; NaHCO_3_, 0.05; NaBr, 0.15; FeCl_3_·6H_2_O, 0.005; hydrocarbons, 30; yeast extract, 3; malt extract, 3; protease peptone, 5] under continuous shaking (120 rpm) at 32 °C and pH 7.2 with 2% (v/v) initial inoculum. All values were taken in triplicates and the average ± SE was recorded after 11 days of incubation. One way ANOVA has been carried out for each row with the P value of 0.05. Bonferroni's post-test shows the DCW was significantly different (*P* < 0.05) against growth (OD at 540 nm) while growth and DCW were not significant (*P* > 0.05) against EPS production.

### 3.6. Influence of nitrogen source

The most common nitrogen sources currently used for EPS production by halophiles include ammonium sulfate, peptone, sodium nitrate, urea and yeast extract but the presence of organic nitrogen sources are known to promote both growth rate and the EPS production [22]. From the results as illustrated in Table 4, it was evident that all the organic nitrogen sources like peptone, yeast extract, casein hydrolysate, beef extract, tryptone have positive influence on the EPS synthesis. In presence of organic nitrogen sources, the isolate was capable of accumulating remarkable amounts of EPS (5.50–6.56 g/L) in the medium concomitant with consumption of glucose. However, amongst the different organic nitrogen source, casein hydrolysate was most preferred one which led to the production of 6.56 g/L of EPS. This may be due to vitamins and cofactors present in organic nitrogen sources which could have played the key role in inducing growth and EPS production [23]. However, during growth in media containing inorganic nitrogen sources, EPS production by the isolate was affected significantly.

**Figure 6.**
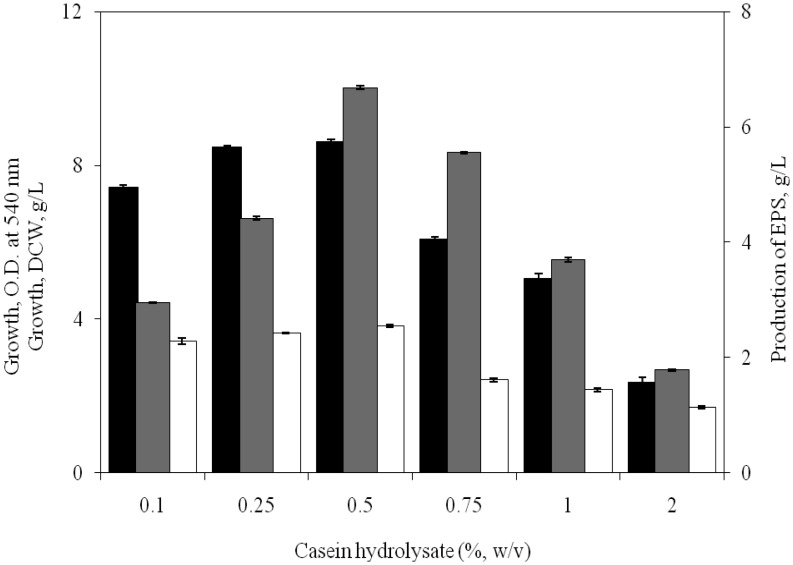
Influence of different concentrations of casein hydrolysate on growth [OD at 540 nm (▪), DCW (□)] and EPS production (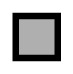) by *H. xianhensis* SUR308. Fermentations were carried out in MY medium [(g/L): NaCl, 25; MgCl_2_·6H_2_O, 9; MgSO_4_·7H_2_O, 13; CaCl_2_·2H_2_O, 0.2; KCl, 1.3; NaHCO_3_, 0.05; NaBr, 0.15; FeCl_3_·6H_2_O, 0.005; glucose, 30; yeast extract, 3; malt extract, 3] under continuous shaking (120 rpm) at different concentrations of casein hydrolysate (1–20 g/L) at 32 °C and pH 7.5 with 2% (v/v) initial inoculum. All values were taken in triplicates and the average ± SE was recorded after 11 days of incubation.

To find out the most suitable concentration of casein hydrolysate for EPS production the isolate was grown in media containing 0.1–2% of casein hydrolysate, other conditions of growth were same as in previous experiments. It was found that EPS production by the isolate raised to 6.68 g/L when the medium was supplemented with 0.5% casein hydrolysate (Figure 6). At concentrations beyond 0.5%, casein hydrolysate was inhibitory to both growth and EPS production.

**Table 4.** Influence of nitrogen sources on growth and EPS production by *H. xianhensis* SUR308.

Nitrogen source	Growth		Production of EPS, g/L
	OD at 540 nm	DCW, g/L	
Control	7.71 ± 0.06	3.04 ± 0.02	5.82 ± 0.01
Tryptone	6.27 ± 0.06	3.32 ± 0.02	5.80 ± 0.01
Beef extract	8.13 ± 0.12	4.51 ± 0.01	5.50 ± 0.01
Casein hydrolysate	8.52 ± 0.03	4.37 ± 0.02	6.56 ± 0.00
Yeast extract	7.71 ± 0.06	3.72 ± 0.00	6.49 ± 0.01
Ammonium sulfate	4.53 ± 0.02	1.81 ± 0.01	2.04 ± 0.01
Ammonium nitrate	0.32 ± 0.02	0.31 ± 0.01	1.51 ± 0.02
Sodium nitrate	1.05 ± 0.10	0.72 ± 0.02	1.52 ± 0.02
Potassium nitrate	0.633 ± 0.03	0.63 ± 0.03	1.23 ± 0.01
Ammonium chloride	2.28 ± 0.03	0.68 ± 0.01	2.40 ± 0.01

Fermentations were carried out in MY medium [(g/L): NaCl, 25; MgCl_2_·6H_2_O, 9; MgSO_4_·7H_2_O, 13; CaCl_2_·2H_2_O, 0.2; KCl, 1.3; NaHCO_3_, 0.05; NaBr, 0.15; FeCl_3_·6H_2_O, 0.005; glucose, 30; yeast extract, 3; malt extract, 3; protease peptone, 5] under continuous shaking (120 rpm) at 32 °C and pH 7.2 with 2% (v/v) initial inoculum. All values were taken in triplicates and the average ± SE was recorded after 11 days of incubation. One way ANOVA has been carried out for each row with the P value of 0.05. Bonferroni's post-test shows the growth (OD at 540 nm), DCW, production of EPS were not significantly different (*P* > 0.05).

### 3.7. Influence of initial inoculum dose

**Figure 7.**
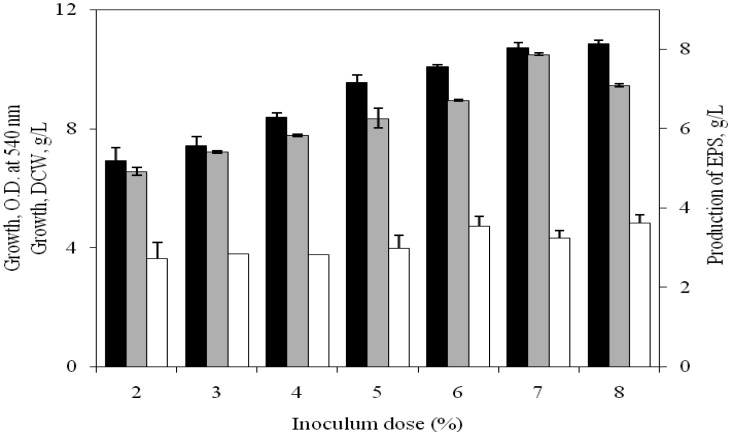
Influence of initial inoculum dose on growth [OD at 540 nm (▪), DCW (□)] and EPS production (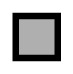) by *H. xianhensis* SUR 308. Fermentations were carried out in MY medium [(g/L): NaCl, 25; MgCl_2_·6H_2_O, 9; MgSO_4_·7H_2_O, 13; CaCl_2_·2H_2_O, 0.2; KCl, 1.3; NaHCO_3_, 0.05; NaBr, 0.15; FeCl_3_·6H_2_O, 0.005; glucose, 30; yeast extract, 3; malt extract, 3; casein hydrolysate, 5] at different inoculum dose of 2–8% (v/v) under continuous shaking (120 rpm) at 32 °C and pH 7.2. All values were taken in triplicates and the average ± SE was recorded after 11 days of incubation.

Since the initial inoculum added to the medium is known to influence the EPS production, MY medium supplemented with 2.5% NaCl, 3% glucose, 0.5% casein hydrolysate at pH 7.5 and with CVF of 1.5:10 was inoculated with freshly grown culture of the isolate at 1–8% (v/v) level and incubated at 32 °C under continuous shaking (120 rpm). As shown in the Figure 7, it was found that the isolate produced 7.87 g/L of EPS when the medium was inoculated with 7% initial inoculum (Figure 7). At this stage, the culture density (OD) turned so thick and prevented normal shaking of the medium. To extract the EPS, the culture medium was initially diluted to separate the cell mass by centrifugation.

### 3.8. Influence of heavy metals

Bacteria resistant to metals often showed production of high amount of exopolysaccharides at heavy metal stressed growth condition [24],[25]. Studies on the heavy metal binding capacity of EPS produced by marine bacteria have proposed that this might represent a survival strategy by reducing their exposure to toxic metals [26]. Heavy metals like Co, Cu, Zn, Ni are essential for metabolism of microorganisms, since they provide vital cofactors for metallo-proteins and enzymes [27].

**Table 5.** Effect of heavy metals on the growth and EPS production by *H. xianhensis* SUR308.

Heavy metal	Concentration, mM	Growth		Production of EPS (g/L)
		OD at 540 nm	DCW, g/L	
Mn	2	10.00 ± 0.01	3.58 ± 0.02	4.93 ± 0.01
	4	10.01 ± 0.00	4.12 ± 0.09	5.68 ± 0.02
	6	10.01 ± 0.00	3.87 ± 0.03	5.70 ± 0.00
	8	10.12 ± 0.00	4.18 ± 0.05	6.75 ± 0.02
Pb	2	3.20 ± 0.00	1.02 ± 0.02	3.15 ± 0.01
	4	1.05 ± 0.00	0.54 ± 0.08	1.13 ± 0.01
	6	0.71 ± 0.01	0.22 ± 0.01	0.91 ± 0.00
Ni	2	2.93 ± 0.02	0.86 ± 0.07	1.37 ± 0.00
	4	7.34 ± 0.03	3.59 ± 0.06	2.34 ± 0.00
Cr	2	10.56 ± 0.11	4.25 ± 0.02	6.95 ± 0.21
Control	-	10.34 ± 0.11	4.28 ± 0.09	6.88 ± 0.25

Fermentations were carried out in MY medium [(g/L): NaCl, 25; MgCl_2_·6H_2_O, 9; MgSO_4_·7H_2_O, 13; CaCl_2_·2H_2_O, 0.2; KCl, 1.3; NaHCO_3_, 0.05; NaBr, 0.15; FeCl_3_·6H_2_O, 0.005; glucose, 30; yeast extract, 3; malt extract, 3; casein hydrolysate, 5] under continuous shaking (120 rpm) at 32 °C and pH 7.2 with 2% (v/v) initial inoculum. All values were taken in triplicates and the average ± SE was recorded after 11 days of incubation. One way ANOVA has been carried out for each row with the P value of 0.05. Bonferroni's post-test shows that the growth (OD at 540 nm), DCW and production of EPS were significantly different (*P* < 0.05) against different concentrations of manganese while were not significantly different against different concentrations lead, nickel and chromium (*P* > 0.05) in the medium.

**Table 6.** Comparative account on the production of exopolysaccharides by different *Halomonas* strains so far available.

*Halomonas* spp.	Optimum l conditions for production	EPS produced	References
*H. alkaliantarctica* strain CRSS	Medium B with maltose and 7.5% NaCl	2.9 g/g	Poli et al., 2004 [28]
*H. alkaliphila*	Medium 2 with 1% Glucose and 10% NaCl	ND	Romano et al., 2006 [29]
*H. almeriensis* MS^T^	MY medium with 1% Glucose and 7.5% NaCl	1.7 g/L	Llamas et al., 2012 [30]
*H. anticariensis*	MY medium with 1% Glucose and 7.5% NaCl	0.3–0.5 g/L	Mata et al., 2006 [9]
*H. eurihalina* F2–7	MY medium with 1% Glucose and 7.5% NaCl	1.4 g/L	Quesada et al., 1993 [4]
*H. eurihalina* Al–12^T^		2.8 g/L
*H. maura* S-30	MY medium with 1% Glucose and 2.5% NaCl	3.8 g/L	Arias et al., 2003 [20]
*H. rifensis*	MY medium with 1% Glucose and 7.5% NaCl	ND	Amjres et al., 2011 [10]
*H. smyrensis*	Medium B with Glucose and 10% NaCl	ND	Poli et al., 2013 [31]
*H. stenophila*	MY medium with 1% Glucose and 5% NaCl	3.89 g/L	Amjres et al., 2015 [32]
*H. ventosae* Al-12^T^	MY medium with 1% Glucose and 7.5% NaCl	0.28 g/L	Mata et al., 2006 [9]
*H. ventosae* Al-16		0.30 g/L
*Halomonas sp*. AAD6	Chemically defined medium with 5% sucrose and 13.5% NaCl	1.073 g/L	Poli et al., 2009 [33]

Addition of toxic heavy metals in the growth medium showed inhibitory effect on growth and EPS production by the isolate SUR308. Among the metals tested manganese (2–8 mM) did not affect growth as well as EPS production (4.93–6.75 g/L), while lead and nickel inhibited growth and EPS production remarkably. However, induction of EPS production by the isolate was observed when the medium was amended with 2 mM chromium. The highest (6.95 g/L) EPS production was observed in 2 mM chromium supplemented medium (Table 5).

## 4. Conclusions

The present study demonstrated the optimization of media composition along with environmental parameters to scale up the production of EPS by *H. xianhensis* SUR308 at laboratory scale. When the EPS production of *H. xianhensis* SUR308 were compared with different strains so far available (Table 6) it showed the highest level of production. Such a significant improvement of EPS production (from 2.58 g/L to 7.87 g/L) was achieved through the selection of glucose, casein hydrolysate and initial inoculum concentration. The EPS production at 32 °C, pH of 7.5 and aeration at 1.5:10 were established as the optimal environmental conditions which could be useful for the large scale production. Furthermore, the isolate was able to produce remarkable amount of EPS in chromium and manganese supplemented medium.

## References

[1] Vandamme EJ, De Baets S, Steinbuchel A (2002). Biopolymers, Polysaccharides I: Polysaccharides from Prokaryotes.

[2] Wan-Mohtar WAAQ, Young L, Abbott GM (2016). Antimicrobial properties and cytotoxicity of sulfated (1,3)-β-D-Glucan from the Mycelium of the mushroom *Ganodermalucidum*. J Microbiol Biotechnol.

[3] Margesin R, Schinner F (2001). Potential of halotolerant and halophilic microorganisms for biotechnology. Extremophiles.

[4] Quesada E, Bejar V, Calvo C (1993). Exopolysaccharide production by *Volcaniella eurihalina*. Experientia.

[5] Bouchotroch S, Quesada E, del Moral A (2001). *Halomonas maura* sp. nov., a novel moderately halophilic, exopolysaccharide-producing bacterium. Int J Syst Evol Microbiol.

[6] Calvo C, Ferrer MR, Martinez-Checa F (1995). Some rheological properties of the extracellular polysaccharide produced by *Volcaniella eurihalina* F2-7. Appl Biochem Biotechnol.

[7] Calvo C, Martinez-Checa F, Mota A (1998). Effect of cations, pH and sulfate content on the viscosity and emulsifying activity of the *Halomonas eurihalina* exopolysaccharide. J Ind Microbiol Biotechnol.

[8] Martinez-Checa F, Bejar V, Martinez-Canovas MJ (2005). *Halomonas almeriensis* sp. nov., a moderately halophilic, exopolysaccharide-producing bacterium from Cabo de Gata, Almeria, south-east Spain. Int J Syst Evol Microbiol.

[9] Mata JA, Bejar V, Llamas I (2006). EPS produced by the recently described halophilic bacteria *Halomonas ventosae* and *Halomonas anticariensis*. Res Microbiol.

[10] Amjres H, Bejar V, Quesada E (2011). *Halomonas rifensis* sp. nov., an exopolysaccharide-producing, halophilic bacterium isolated from a solar saltern. Int J Syst Evol Microbiol.

[11] Llamas I, Bejar V, Martinez-Checa F (2011). *Halomonas stenophila* sp. nov., a halophilic bacterium that produces sulphate exopolysaccharides with biological activity. Int J Syst Evol Microbiol.

[12] Biswas J, Mandal S, Paul AK (2015). Production, partial purification and some bio-physicochemical properties of EPS produced by *Halomonas xianhensis* SUR308 isolated from a saltern environment. J Biol Active Pdts Nat.

[13] Biswas J, Ganguly J, Paul AK (2015). Partial characterization of an extracellular polysaccharide produced by a moderately halophilic bacterium *Halomonas xianhensis* SUR308. Biofouling.

[14] Ventosa A, Quesada E, Rodriguez-Valera F (1982). Numerical taxonomy of moderately halophilic gram-negative rods. J Gen Microbiol.

[15] Dubois M, Gilles KA, Hamilton JK (1956). Colorimetric method for determination of sugars and related substances. Anal Chem.

[16] Miller GL (1972). Use of DNS reagent for the determination of glucose. Anal Chem.

[17] Bejar V, Calvo C, Moliz J (1996). Effect of growth conditions on the rheological properties and chemical composition of *Volcaniella eurihalina* exopolysaccharide. Appl Biochem Biotechnol.

[18] Cerning J, Renard CMGC, Thibault JF (1994). Carbon source requirements for exopolysaccharide production by *Lactobacillus casei* CG11 and partial structure analysis of the polymer. Appl Environ Microbiol.

[19] Rehm BHA (2009). Microbial production of biopolymers and polymer precursors: applications and perspectives.

[20] Arias S, del Moral A, Ferrer MR (2003). Mauran, an exopolysaccharide produced by the halophilic bacterium *Halomonas maura*, with a novel composition and interesting properties for biotechnology. Extremophiles.

[21] Martinez-Checa F, Toledo FL, El Mabrouki K (2007). Characteristics of bioemulsifier V2-7 synthesized in culture media added of hydrocarbons: chemical composition, emulsifying activity and rheological properties. Bioresource Technol.

[22] Farres J, Caminal G, Lopez-Santin J (1997). Influence of phosphate on rhamnose-containing exopolysaccharide rheology and production by *Klebsiella* I-174. Appl Microbiol Biotechnol.

[23] Abe K, Hayashi H, Maloney PC (1996). Exchange of aspartate and alanine mechanism for development of a proton-motive force in bacteria. J Biol Chem.

[24] Kim SY, Kim JH, Kim CJ (1996). Metal adsorption of the polysaccharide produced from *Methylobacterium organophilum*. Biotechnol Lett.

[25] Kazy SK, Sar P, Singh SP (2002). Extracellular polysaccharides of a copper-sensitive and a copper-resistant *Pseudomonas aeruginosa* strain: synthesis, chemical nature and copper binding. Word J Microbiol Biotechnol.

[26] Loaec M, Olier R, Guezennec J (1998). Chelating properties of bacterial polysaccharides from deep-sea hydrothermal vents. Carbohyd Polym.

[27] Hassen N, Saidi M, Cherif M (1998). Effects of heavy metals on Pseudomonas aeruginosa and Bacillus thuringiensis. Bioresource Technol.

[28] Poli A, Moriello VS, Esposito E (2004). Exopolysaccharide production by a new *Halomonas* strain CRSS isolated from saline lake Cape Russell in Antarctica growing on complex and defined media. Biotechnol Lett.

[29] Romano I, Lama L, Nicolaus B (2006). *Oceanobacillus oncorhynchi* subsp. *Incaldanensis* subsp. nov., an alkalitolerant halophile isolated from an algal mat collected from a sulfurous spring in Campania (Italy), and emended description of *Oceanobacillus oncorhynchi*. Int J Syst Evol Microbiol.

[30] Llamas I, Amjres H, Mata JA (2012). The potential biotechnological applications of the exopolysaccharide produced by the halophilic bacterium *Halomonas almeriensis*. Molecules.

[31] Poli A, Nicolaus B, Denizci AA (2013). *Halomonas smyrnensis* sp. nov., a moderately halophilic, exopolysaccharide-producing bacterium. Int J Syst Evol Microbiol.

[32] Amjres H, Bejar V, Quesada E (2015). Characterization of haloglycan, an exopolysaccharide produced by *Halomonas stenophila* HK30. Int J Biol Macromol.

[33] Poli A, Kazak H, Gurleyendag B (2009). High level synthesis of levan by a novel *Halomonas* species growing on defined media. Carbohyd Polym.

